# Root-Zone Heating Boosts the Production of Mini Romaine Lettuce Grown in Nutrient Film Technique and Aeroponics Systems

**DOI:** 10.3390/plants15030422

**Published:** 2026-01-30

**Authors:** Filippos Bantis, Nikolaos Tostsidis, George Zervoudakis, Athanasios Koukounaras, Athanasios Koulopoulos

**Affiliations:** 1 Department of Agriculture, University of Western Macedonia, 53100 Florina, Greece; 2 Department of Agriculture, University of Patras, 30200 Messolonghi, Greece; up1082677@ac.upatras.gr (N.T.); gzerv@upatras.gr (G.Z.); tkoulop@upatras.gr (A.K.); 3 School of Agriculture, Aristotle University of Thessaloniki, 54124 Thessaloniki, Greece; thankou@agro.auth.gr

**Keywords:** soilless systems, hydroponics, NFT, root-zone temperature, nutrient solution

## Abstract

Root-zone temperature is a critical environmental parameter affecting the development, physiology, and nutritional status of leafy vegetables in soilless systems such as the nutrient film technique (NFT) and aeroponics. In the present article, we report on responses of mini Romaine lettuce (*Lactuca sativa* L.) upon cultivation using heated nutrient solution targeting minimum temperatures of 14, 18, and 22 °C versus ambient (control; 11–12 °C), both in the NFT and in the aeroponics system. In both systems, the higher temperatures (i.e., 18 and 22 °C) led to considerably higher leaf mass per system area (127–232% in NFT; 54–75% in aeroponics) and leaf length (more than 21% in all cases). Root dry weight and total soluble solids were positively affected by increasing temperatures only in the NFT. Performance indices of the photosynthetic mechanism (PI_abs_ and PI_tot_) were increased in the lower temperatures in the NFT. Antioxidant activity and total phenolics were not affected in either soilless system. Total chlorophylls and carotenoids were enhanced by 18 and 22 °C in the NFT and aeroponics, respectively, while anthocyanins were also variably affected. Finally, nitrate content was significantly reduced (−42%) in 18 °C in the NFT. Sub-optimal root-zone temperatures constrained root development and biomass accumulation, indicating that growth limitation was mainly driven by sink-related processes rather than carbon assimilation. Overall, heating the nutrient solution to a minimum of 22 °C in low- and mid-tech greenhouses during cool months can increase the production efficiency of mini Romaine lettuce in the NFT and aeroponics.

## 1. Introduction

Mini leafy vegetables show a continuously increasing demand due to their high nutritional content, excellent taste, tender texture, and convenient nature when it comes to ready-to-eat vegetables [1]. Mini or miniature is an intermediate stage where heads are harvested at a size between the baby leaf and the mature type. This is a common practice in hydroponic systems and soil-based experiments due to its compact size, short cultivation cycle, and suitability for controlled-environment agriculture [2,3]. In particular, lettuce (*Lactuca sativa* L.) is among the most important leafy vegetables cultivated worldwide until mini stage, constituting a specialty type of lettuce. The crop shows elevated economic and nutritional value due to its high demand for immediate consumption as fresh-cut salads. Lettuce holds a special place in the Mediterranean diet since it is a key ingredient in salads and is related to the consumption of fresh, light, and nutritious meals. In many countries, lettuce covers large areas and contributes significantly to farmers’ income and food security. Increased emphasis on matters related to food quality, high nutritional content, and sustainable production processes makes lettuce a highly essential food as well as a strategically important vegetable product. Lettuce cultivation shows high adaptability to various production models, from outdoor fields to greenhouses and vertical farms, and from soil to hydroponic systems, thus constituting it as a product with high market availability. The Romaine (or Cos; var. Longifolia) type in particular is characterized by elongated leaves, crispy texture, and overall high nutritional content; thus, it is commercially very popular [4]. On a worldwide scale, lettuce yield surpasses 28 million tons, with China being the leading producer [5]. In Europe, the leading producer countries are Spain, Italy, and France, which export large quantities mainly within Europe [5].

Soilless (hydroponic) cultivation of lettuce nowadays is gaining increasing attention. The cultivation of leafy vegetables in hydroponic systems is considered one of the most efficient strategies of intensive food production employed in confined environments (i.e., greenhouses and vertical farms) under controlled conditions. Due to the increasing demand for fresh, safe, high-quality food, soilless production systems are crucial for the future of agriculture, especially in regions with limited water and land availability and high urbanization [6]. Within hydroponic production, the potential of precise root-environment control offers substantial benefits over traditional soil agriculture, which shows significant nutrient losses and an inadequate ecological footprint. Among the various soilless systems, the nutrient film technique (NFT) and aeroponics allow for precise control of the root zone environment, high water- and nutrient-use efficiency, and an enhanced overall capacity for cultivation under relatively controlled conditions [6]. Briefly, in the NFT, a thin layer of nutrient solution flows through a horizontal canal where the root system directly receives water and nutrients [7]. Aeroponics can be described as a soilless system where roots are exposed in a tube (usually vertically placed) and periodically moisturized with nutrient solution [8]. Both systems vary considerably with respect to the environment where the root system is exposed. The discrepancies between soilless systems emphasizes the need for targeted management strategies depending on the cultural characteristics of each system. Moreover, soilless cultivation is considered a tool critical to achieving the Sustainable Development Goals established by the United Nations, involving the cultivation of traditional or underutilized leafy vegetables [9], as well as the implementation of organically produced compounds such as biostimulants and liquid digestates, among others [10,11]. The success of soilless cultivation largely relies on the stability of critical parameters such as root-zone temperature and air temperature [12].

Nowadays, root-zone temperature control is a hot topic if we take into account the adaptation of agricultural production to the climate crisis. The extreme temperature fluctuations projected in the following decades will require the development of strategies to control and stabilize the root-zone microenvironment in a similar manner as the air environment. The use of novel technologies such as optimized soilless systems, real-time sensors, and precision-heating systems will be essential parts of sustainable and efficient crop production. Root-zone temperature not only alters the root system growth and architecture affecting root cell elongation and division [13,14] but also affects several physiological processes, including nutrient absorption and distribution, enzyme activity, transpiration, photosynthesis [15], reactive oxygen species metabolism, plasma membrane structure, and osmotic balance [16]. A study with lettuce and rocket baby leaves revealed enhanced productivity when root-zone heating was applied during winter production [17]. Moreover, root-zone temperature is often overlooked since most attention is given to aerial parameters (i.e., temperature, relative humidity, light, CO_2_ etc.). Several articles focused on the optimum air temperature for lettuce, which was defined in the range of 17–28 °C during the day and 3–12 °C during the night [18]. In a low- or mid-tech greenhouse with an absence of heating systems, cold conditions (i.e., winter months) can be very challenging, decreasing the nutrient solution temperature to suboptimal levels and leading to decelerated or even halted development, biomass decrease, and alterations in plant nutritional content [19]. For example, red leaf lettuce showed reduced leaf area, stem diameter, and shoot and root fresh weight when the root system was exposed to 10 °C [20]. Root-zone temperatures of approximately 18–22 °C are generally considered optimal for lettuce growth in soilless systems, supporting efficient root metabolism, nutrient uptake, and balanced shoot development. The abovementioned constraints may also negatively affect the taste and marketability of produced crops [21]. On a practical level, root-zone temperature control can be an essential tool for optimized production efficiency, especially during the colder months, in low- to mid-tech greenhouses where environmental conditions are characterized by sharp fluctuations. The difference between maximum and minimum, also known as the diurnal temperature range (ΔT), is a critical index for explaining various developmental and physiological observations. Smaller fluctuations can be correlated with better balance between biomass production and other qualitative characteristics.

Chlorophyll fluorescence parameters, including PI_abs_ (performance index on absorption basis) and PI_tot_ (total performance index), reflect the overall functionality of photosystem II (PSII) and photosynthetic performance under different thermal conditions [22]. Specifically, PI_abs_ shows the overall functionality of PSII and the efficiency with which absorbed light energy is converted into photochemical processes, while PI_tot_ extends this assessment to the combined performance of both PSII and PSI [23]. These indices respond rapidly to environmental constraints, including sub-optimal root-zone temperature, and therefore provide an effective means of detecting early impairments in the photosynthetic apparatus. Nitrate concentration was assessed as a key indicator of nitrogen metabolism due to its sensitivity to root-zone temperature. Moreover, nitrate content is an indicator of product quality linked with consumer safety under current EU regulations [17]. In addition, photosynthetic pigments (i.e., chlorophylls and carotenoids) and secondary metabolites (e.g., phenolics, such as anthocyanins) are excellent indicators of photosynthetic capacity and photoprotection, usually associated with stress responses such as low temperature [24].

To that end, our objective was to test the effect of heated root-zone temperature on mini Romaine lettuce growth, physiological attributes, and nutritional content, upon cultivation in an NFT and an aeroponics system. We hypothesized that (i) root-zone temperatures above 18 °C would significantly increase leaf mass per system area (LMSA) and morphological attributes compared to the non-heated control, and (ii) sub-optimal root-zone temperatures below 14 °C would limit growth parameters in both soilless systems. The literature usually deals with comparing normal with extremely high (e.g., 35 °C) or low temperatures (e.g., 10 °C) [19,25]. In our case, temperature regimes starting from a minimum of 14, 18, and 22 °C were compared to a respective non-treated control treatment to identify the minimum required nutrient solution temperature within a normal non-stressful range for optimum plant growth, physiological status, and nutritional quality during cultivation in a mid-tech greenhouse in winter months. According to the above, the NFT treatments are hereby labeled as NFT-C, NFT-14, NFT-18, and NFT-22, while the aeroponics treatments are hereby labeled as Aero-C, Aero-14, Aero-18, and Aero-22. It should be noted that the two soilless systems were not directly compared to each other but were selected to examine the responses of root-zone heating and showcase possible discrepancies within each system.

## 2. Results

### 2.1. Growth and Biomass Parameters

**Figure 1:**
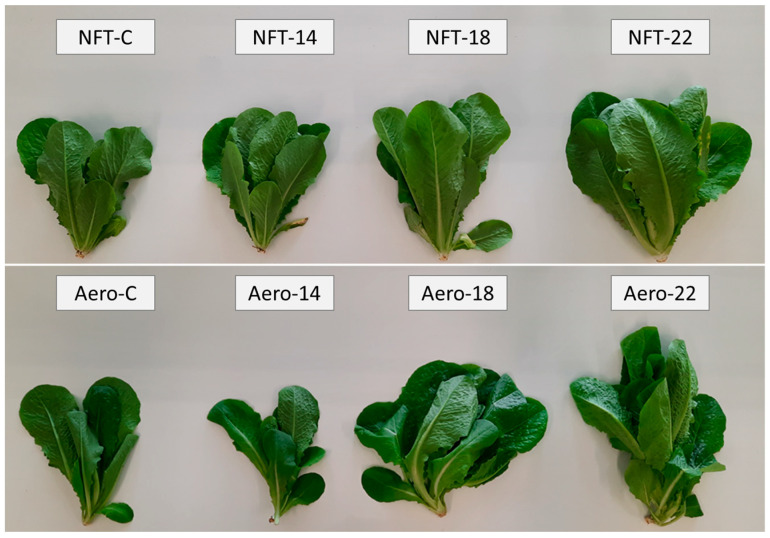
Representative images at harvest of mini Romaine lettuce cultivated in NFT (nutrient film technique) or Aero (aeroponics) soilless systems and treated with different root-zone temperatures in the nutrient solutions. C: control (mean minimum temperature of 12.2 and 10.9 °C for NFT and Aero, respectively).

**Figure 2:**
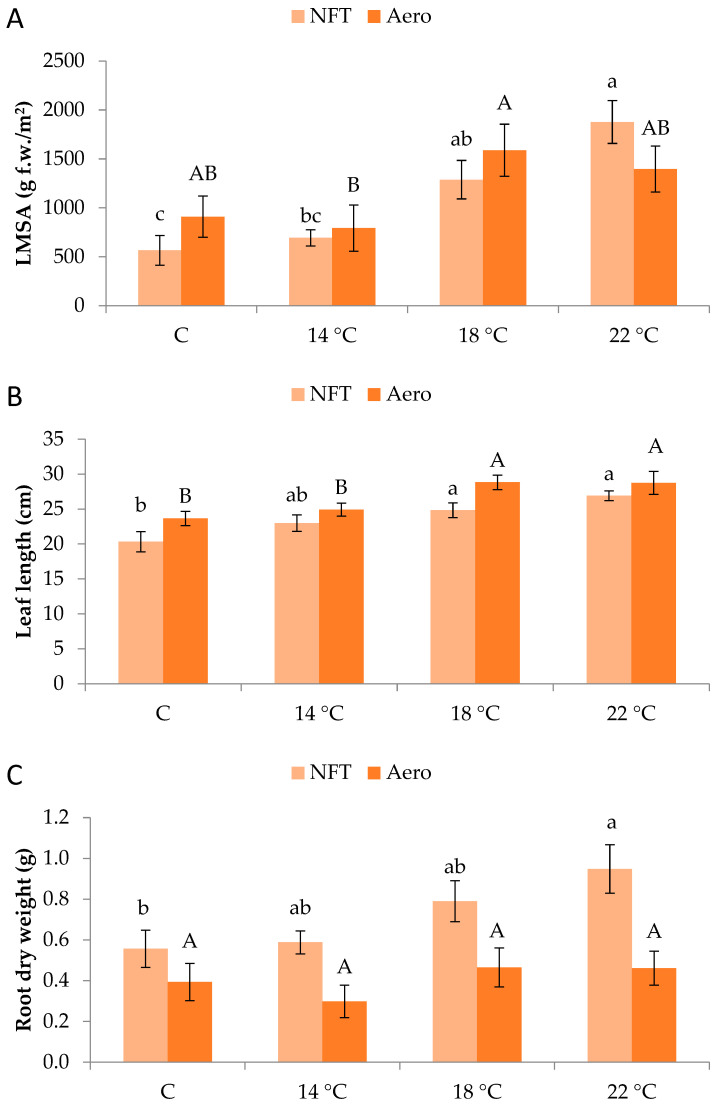
(**A**) Leaf mass per system area (LMSA), (**B**) leaf length, and (**C**) root dry weight of mini Romaine lettuce cultivated in NFT (nutrient film technique) or Aero (aeroponics) soilless systems and treated with different root-zone temperatures in the nutrient solutions. LMSA represents leaf fresh mass per unit growing area, adjusted for the plant density of each soilless system (i.e., 15.2 plants/m^2^ in the NFT and 22.2 plants/m^2^ in Aero). Within each soilless system, bars (N = 7 for NFT; N = 8 for Aero; ± SE) followed by different lowercase (NFT) or capital (Aero) letters are significantly different (*p* ≤ 0.05). C: control (mean minimum temperature of 12.2 and 10.9 °C for NFT and Aero, respectively). No direct statistical comparison between the soilless systems was conducted.

Plants grew normally during the experimental period. No physiological or phytopathological disorders were observed (Figure 1). LMSA was significantly affected by root zone temperature. In the NFT, LMSA was greater at NFT-22 compared to NFT-C and NFT-14, while NFT-18 was greater than NFT-C (*p* < 0.001). In aeroponics, LMSA was significantly greater in Aero-18 compared to Aero-14 (*p* = 0.041) (Figure 2A). Leaf length in the NFT was significantly greater in NFT-22 and NFT-18 compared to NFT-C (*p* = 0.004). Quite similarly, in aeroponics, leaf length was greater in Aero-22 and Aero-18 compared to Aero-C and Aero-14 (*p* = 0.010) (Figure 2B). In the NFT, root dry weight was significantly greater in NFT-22 compared to NFT-C (*p* = 0.027). On the contrary, root dry weight was not affected by temperature in aeroponics (Figure 2C).

### 2.2. Physiological Parameters

**Figure 3:**
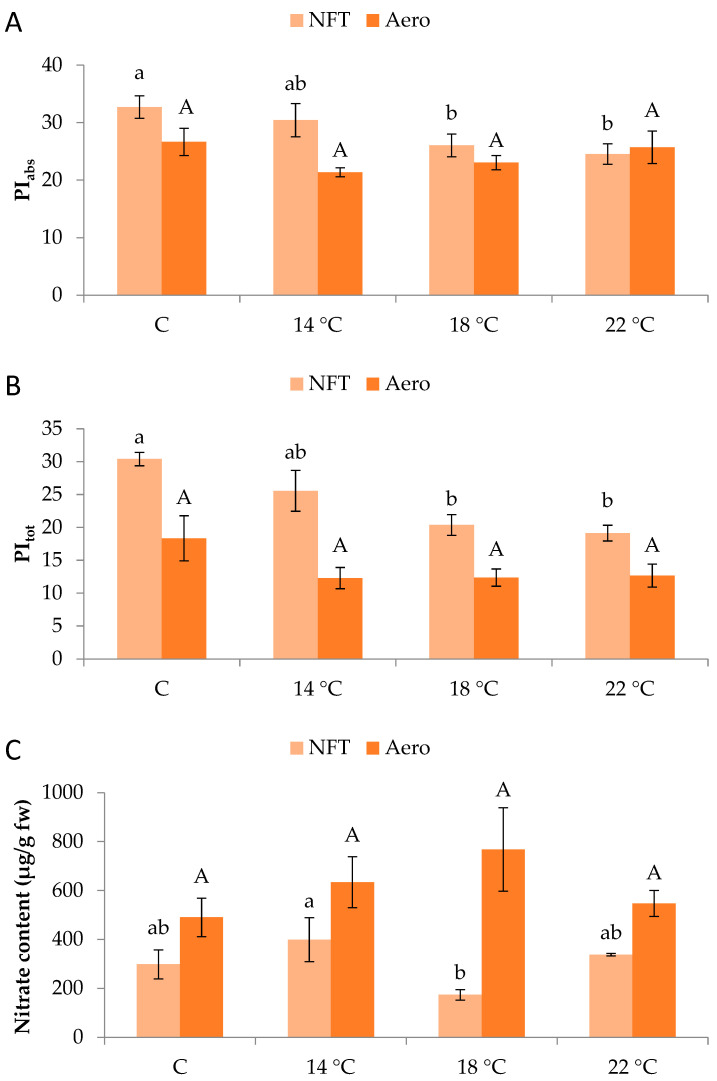
(**A**) PI_abs_, (**B**) PI_tot_, and (**C**) nitrate content of mini Romaine lettuce cultivated in NFT (nutrient film technique) or Aero (aeroponics) soilless systems and treated with different root-zone temperatures in the nutrient solutions. PI_abs_: performance index on absorption basis (PSII performance); PI_tot_: total performance index integrating PSII and PSI functionality. Within each soilless system, bars (N = 7 for NFT; N = 8 for Aero; ± SE, or N = 3 for nitrate content) followed by different lowercase (NFT) or capital (Aero) letters are significantly different (*p* ≤ 0.05). C: control (mean minimum temperature of 12.2 and 10.9 °C for NFT and Aero, respectively). No direct statistical comparison between the soilless systems was conducted.

Regarding chlorophyll fluorescence parameters, PI_abs_ and PI_tot_ in the NFT were significantly greater in NFT-C compared to NFT-18 and NFT-22 (*p* = 0.045 and *p* = 0.001, respectively). No significant effects were recorded in aeroponics (Figure 3A,B).

### 2.3. Nutritional Safety and Quality

In the NFT, greater nitrate content was found in NFT-14 than NFT-18 (*p* = 0.047), while different temperatures did not impose specific effects in aeroponics (Figure 3C). Nitrate concentrations reached 768 mg/kg of fresh weight across all treatments and remained below the maximum limit established for lettuce by the European Commission (5000 mg/kg of fresh weight).

**Figure 4:**
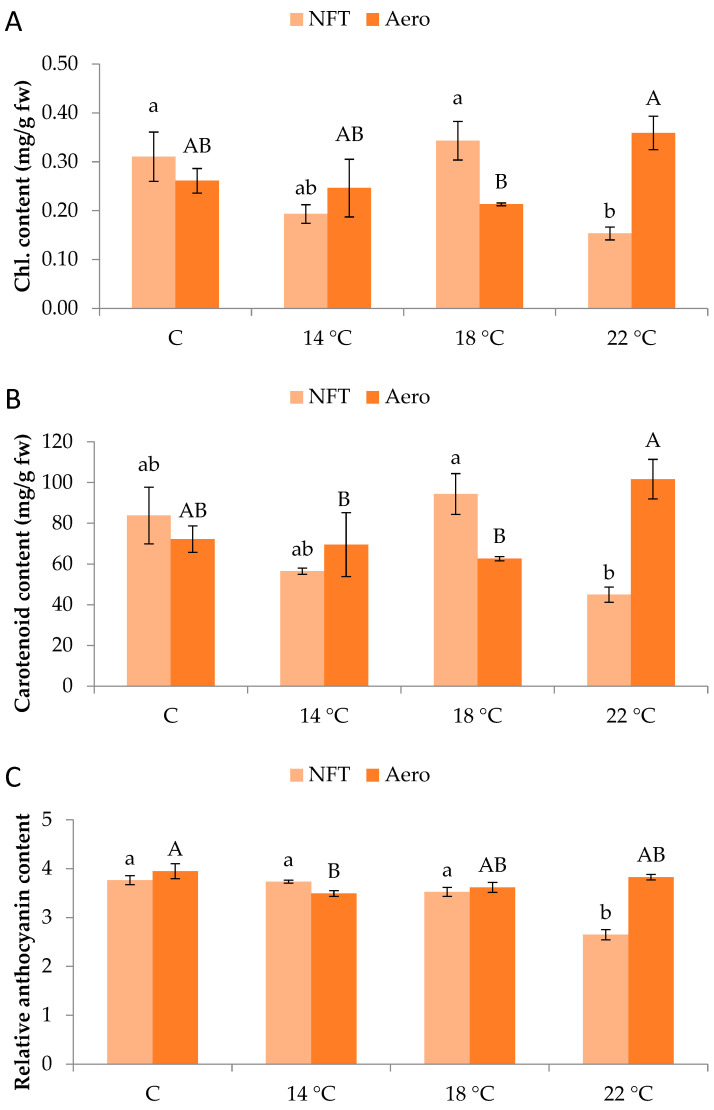
(**A**) Total chlorophyll content, (**B**) total carotenoid content, and (**C**) relative anthocyanin content of mini Romaine lettuce cultivated in NFT (nutrient film technique) or Aero (aeroponics) soilless systems and treated with different root-zone temperatures in the nutrient solutions. Within each soilless system, bars (N = 3 ± SE, or N = 6 for anthocyanins) followed by different lowercase (NFT) or capital (Aero) letters are significantly different (*p* ≤ 0.05). C: control (mean minimum temperature of 12.2 and 10.9 °C for NFT and Aero, respectively). Anthocyanin content is expressed as relative index values. No direct statistical comparison between the soilless systems was conducted.

Regarding pigment quantification, total chlorophylls were greater in NFT-18 and NFT-C compared to NFT-22 (*p* = 0.012). On the contrary, in aeroponics, total chlorophylls were greater in Aero-22 than Aero-18 (*p* = 0.042) (Figure 4A). Similar results were obtained for carotenoid content. In the NFT, greater values were recorded in NFT-18 compared to NFT-22 (*p* = 0.013), while in aeroponics, greater values were found in Aero-22 than Aero-18 and Aero-14 (*p* = 0.039) (Figure 4B). In the NFT, relative anthocyanin content was significantly greater in NFT-C, NFT-14, and NFT-18 compared to NFT-22, which showed the lowest values (*p* < 0.001). Regarding aeroponics, relative anthocyanin content was greater in Aero-C compared to Aero-14 (*p* = 0.019) (Figure 4C).

**Figure 5:**
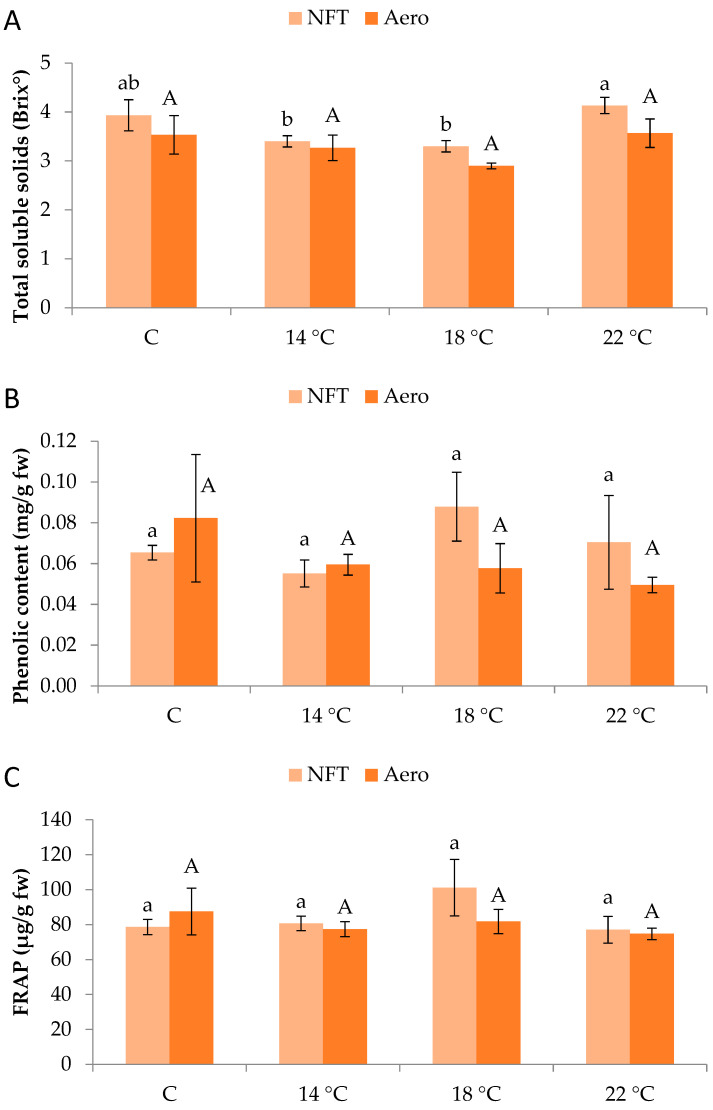
(**A**) Total soluble solids, (**B**) total phenolic content, and (**C**) antioxidant activity (displayed by ferric-reducing antioxidant power-FRAP) of mini Romaine lettuce cultivated in NFT (nutrient film technique) or Aero (aeroponics) soilless systems and treated with different root-zone temperatures in the nutrient solutions. Within each soilless system, bars (N = 3 ± SE) followed by different lowercase (NFT) or capital (Aero) letters are significantly different (*p* ≤ 0.05). C: control (mean minimum temperature of 12.2 and 10.9 °C for NFT and Aero, respectively). No direct statistical comparison between the soilless systems was conducted.

Regarding total soluble solids (Brix°), values were significantly greater in NFT-22 compared to NFT-18 and NFT-14 (*p* = 0.046), while Aeroponics did not show significant differences between temperature regimes (Figure 5A). Moreover, total phenolics content and antioxidant activity (displayed by FRAP) were not significantly affected by temperature, in either the NFT or aeroponics (Figure 5B,C).

## 3. Discussion

Our results help clarify the sensitivity range of the lettuce root system to thermal regimes under soilless cultivation, particularly during winter production. The decline in growth and photosynthetic indices observed under minimum 14 °C indicates that Romaine lettuce exhibits a narrow tolerance window, with root metabolism and hydraulic conductance significantly constrained below approximately 18 °C [15]. These findings contribute to refining species-specific thermal thresholds relevant to controlled-environment production. Moreover, it should be noted that although treatments were defined by minimum root-zone temperatures, plants were exposed to mean root-zone temperatures approaching values (c.f. Table 1) previously reported as optimal for lettuce growth in hydroponic systems, e.g., Ref. [26].

In particular, elevated nutrient solution temperature led to a considerable increase in aboveground characteristics related to leaf biomass, such as LMSA and leaf length in both soilless systems, as well as enhanced root system development in the NFT. Along with minimum temperature, the diurnal temperature range also affects the root activity. In our case, treatments with higher ΔΤ (i.e., control and 14 °C treatments) inhibited plant growth compared to treatments with lower ΔΤ values. In the present study, ΔT was not a controlled factor; thus, the observed association between increased ΔΤ and lower plant growth should be interpreted as correlative. Our findings reaffirm previous results showing the positive effect of about 20 °C root-zone temperature for leafy vegetables’ growth [20,27]. For example, several lettuce cultivars exhibited greater fresh weight under a root-zone temperature of 21.1 °C than ambient temperatures of 18.3 °C and higher in an NFT system [28]. *Eruca sativa* (rocket) plants grown in an aeroponics system under 20 °C root-zone temperature showed considerably greater productivity compared to higher ambient temperatures in the root zone [27]. In another experiment with a deep water culture hydroponic system, lettuce and arugula showed higher yield when grown under elevated root-zone temperature (about 15 °C daily minimum and 20 °C daily average) powered by a photovoltaic system compared to ambient temperature (2–6 °C lower root-zone temperature) during winter [17]. Low root-zone temperatures are known to decelerate enzyme and transport protein activities, limiting water and nutrient absorption, and ultimately inhibiting plant growth [19]. The production of new root tissue includes cell division, enlargement, and differentiation [29]. Root growth depends on the meristem tissue activity, generating new cells that subsequently expand and elongate. Root cell development is influenced by temperature since both the duration of mitosis and the cell-doubling time are shortened when the temperature is increased from 3 to 25 °C [13]. For decades, there have been reports that growth of the length of roots is optimum around 20 °C, decreasing towards 10 °C and 30 °C. Moreover, it is considered that root cell elongation takes place in two steps. The first step should involve a loosening of the cell wall and a cell stretching, while during the second one, a new cell wall formation is conducted by new microfibrils [30]. In the expansion zone of the roots, cells undergo several fold size enlargements that cannot be achieved with the initial primary wall. Secondary wall formation is accompanied by cell enlargement and differentiation, a resource-demanding process. Research data from plants that have been evolutionarily adapted to very low-temperature environments—for example, alpine species—imply that cell differentiation (including lignification) is a likely cause of root growth cessation at very low temperatures that otherwise still enable photosynthesis [29].

In our aeroponics system, root development was similar under all treatments due to specific conditions in the root environment. It should be mentioned that the actual temperature in the root system may have differed slightly from the reservoir temperature, particularly in the aeroponics system due to its lower thermal inertia. However, the controlled nutrient solution temperature remains the primary determinant of root thermal conditions and allows meaningful comparison among treatments. This approach (temperature control in the nutrient solution reservoir) is common in such experiments, e.g., Refs. [15,26]. In particular, in low-pressure aeroponics systems, the roots are exposed to an environment with high humidity but relatively low heat capacity, possibly meaning that root-zone temperature control is difficult to manage with respect to root growth. This suggests that root-zone temperature effects in aeroponics may not necessarily be expressed as changes in root biomass, but may instead be reflected in aboveground growth and physiological responses. On the other hand, root-zone temperature in the NFT can efficiently be adjusted and impose its effects related to the root system development. Quite similarly, in a study involving 17 lettuce cultivars grown in an NFT system, root dry biomass was more pronounced under a 21.1 °C root-zone temperature compared to 18.3 °C and ambient temperature [28]. In another study, red leaf lettuce had greater root dry weight when treated with 25 °C compared to 15 and 35 °C in an NFT [26]. It seems that elevated root-zone temperature facilitates the distribution of photosynthetic assimilates towards the leaves, providing an explanation for the LMSA and leaf length results. In addition, beneficial root-zone temperature may promote the expression of genes related to cell differentiation in roots, which was observed in the NFT but not in aeroponics. For example, previous studies showed that variations in root-zone temperature can alter gene expression profiles in roots, which may lead to changes in root architecture [31,32].

Noticeably, the parameters related to the performance of the photosynthetic apparatus, PI_abs_ and PI_tot_, were higher in the lowest temperature regimes of the NFT. These values remained within the normal range for healthy plants, suggesting that the photosynthetic apparatus maintained functionality under sub-optimal root-zone temperatures. Low or high root-zone temperature can impose an adjusted increase in the photosystem II performance, leading to a temporary enhancement of the photochemical efficiency triggered by environmental stresses [25]. On the other hand, extreme stress in terms of intensity and duration may lead to temporary impairment of the photosynthetic apparatus, as displayed for rocket and lettuce, which were treated with 42 °C root-zone temperature compared to 25 °C [25]. The absence of significant differences in aeroponics indicates the lower sensitivity of this system when it comes to variable temperature regimes. The increase in PI_abs_ and PI_tot_ when plants were exposed to lower root-zone temperatures suggests that plants maintain photosynthetic performance under suboptimal conditions, which may be reflected in the measured aboveground parameters. This possibly explains why NFT plants presented normal levels in both tested indices even in treatments with low-temperature regimes. Earlier studies have indicated that tissue formation becomes very slow below 5 °C and was never observed below 0 °C, a temperature that still permits CO_2_ uptake at ca. 30% of photosynthetic capacity. Hence, it seems that at low temperatures plant growth is not carbon limited. Lettuce plants seem to be able to maintain relatively sufficient rates of photosynthetic carbon assimilation even at low temperatures, while the process of tissue formation (i.e., cell division, elongation, and differentiation) is rather more sensitive. As previously mentioned, plant growth in cold climates is not limited by carbon assimilation (source activity) but rather by reduced carbon investment into new tissues (sink limitation) [29].

Nitrate content was variably affected within the two soilless systems, with no significant differences observed in aeroponics. Quite similarly, Karnoutsos et al. [17] reported no effect of two root-zone temperatures (20 °C versus ambient) in lettuce and arugula grown in deep water culture. However, in the NFT of our experiment, a low temperature of 14 °C led to greater nitrate amounts, possibly due to the limited activity of the nitrate-reducing enzyme nitrate reductase, which is reportedly heat-sensitive [33]. On the other hand, the enhanced nitrate content at low temperature could be the result of increased absorption since it has been mentioned that roots facing short-duration low-temperature stress respond by increasing root water uptake, thus also increasing nutrient absorption [14]. Nitrate accumulation in the leaves of leafy vegetables is a crucial matter for consumers’ safety as well as plant physiology. It should be noted that nitrate content in all soilless systems and temperature regimes was well within the legal amounts defined by the European Commission Regulation No. 1258/2011 for lettuce (up to 5000 mg/kg of fresh weight). The compliance of our samples with the EU regulation under all treatments is very encouraging, as it proves that even in sub-optimum root-zone temperatures, soilless systems can lead to safe products with low nitrate levels. This is particularly important for markets such as in Central and Northern Europe, where temperatures are lower and leafy vegetables tend to produce more nitrates.

The different photosynthetic pigment accumulation observed under each soilless system possibly mirrors the variable microclimatic conditions surrounding the root systems. A study involving *Amaranthus tricolor* revealed higher photosynthetic pigment and bioactive compound content under a root-zone temperature of 20 °C for 7 days [34]. Meanwhile, the reduction in anthocyanins in the higher NFT temperature in our experiment was possibly due to favorable conditions that limit the oxidative stress and the accumulation of secondary metabolites, leading more assimilates towards leaf growth. Such pigments are typically produced as a stress response due to the presence of reactive oxygen species (ROS), while they are associated with enhanced antioxidant enzyme activity [35]. A similar observation but with elevated root-zone temperature was reported for red leaf lettuce, which produced more pigments (i.e., chlorophylls, carotenoids, and anthocyanins) under the higher extreme temperature (35 °C), a typical response to heat stress [26].

Total soluble solids were increased with elevated temperature in the NFT, showcasing the enhanced photosynthetic activity as well as the greater rate of sugar flow. This could be due to more intense photosynthetic activity leading to a greater production of carbohydrates such as sugars, a characteristic linked to higher organoleptic value and greater acceptance by consumers. On the other hand, when plants are exposed to low temperatures, water loss occurs in cells. Hence, the increased soluble solid content of the control treatment compared with the 14 and 18 °C ones could be a result of osmotic adjustment, accumulating various organic substances (or even inorganic ones such as nitrate) to lower the osmotic potential of cells, thus maintaining a certain osmotic concentration in cells [16]. In aeroponics, the effect of temperature was insignificant for total soluble solid accumulation. The same was observed for total phenolics and antioxidant activity (i.e., FRAP) in both soilless systems, indicating that the tested temperature regimes did not impose high oxidative stress to trigger the production and accumulation of protective secondary metabolites. A similar observation was made for lettuce and arugula grown in spring, summer, and winter experiments, when total phenolics and antioxidant activity were not affected by the controlled (about 20 °C) compared to the ambient temperature [17]. On the other hand, a study involving arugula and lettuce (canasta) in aeroponics reported an increase in total phenolic compounds and ascorbic acid at harvest when plants were treated with increased root-zone temperature up to 42 °C [25]. That response was attributed to ROS such as hydrogen peroxide produced due to heat stress, which triggers the accumulation of antioxidant compounds (such as phenolics) to alleviate the adverse effects. It seems that in our case, a significant stress response was not imposed since the tested root-zone temperatures lie within the normal range for optimum growth of lettuce in soilless systems. This is reaffirmed by the PI_abs_ and PI_tot_ values in both soilless systems, indicating healthy plants.

## 4. Materials and Methods

### 4.1. Growth Conditions and Treatments

The experiment took place in a plastic, non-heated greenhouse in Messolonghi, Greece (N 38.366; E 21.476), during the winter of 2023–2024. Romaine lettuce seedlings (cv. Parris Island) were obtained from a certified nursery. Seedlings were produced in peat plugs (3 × 3 cm). Prior to transplantation, peat was gently removed by rinsing the root system with running water to minimize mechanical damage of the soilless systems. All seedlings were visually inspected to ensure uniformity in size, leaf number (four leaves), and root integrity, and placed in plastic net pots. The seedlings were transplanted either to an NFT hydroponic system or an aeroponics system, described below.

**Figure 6:**
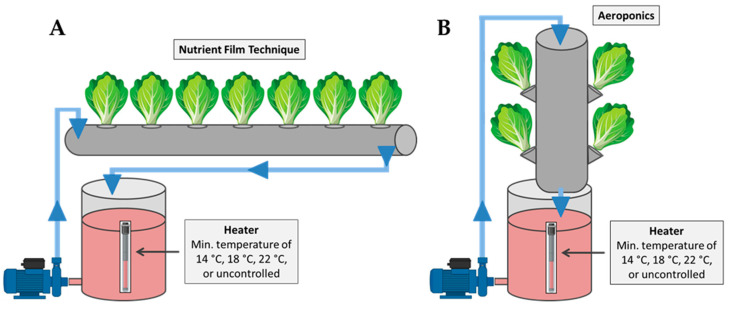
Schematic representation of the experimental setup used for the cultivation of mini Romaine lettuce in the two soilless systems: (**A**) nutrient film technique and (**B**) aeroponics.

The experiment was conducted using eight independent closed-loop hydroponic subsystems operated either as the nutrient film technique (four subsystems) or as aeroponics (four subsystems). All subsystems shared the same electromechanical infrastructure (nutrient solution reservoirs, pumps, temperature-control and monitoring systems), with structural modifications applied to create the distinct growing environments for the NFT and aeroponics. Specifically, the NFT units (Figure 6A) consisted of PVC pipes (100 mm diameter, 1400 mm length) placed horizontally. A nutrient solution reservoir supplied each NFT pipe via a pump (6 W, 60 L h^−1^), delivering a continuous thin film of nutrient solution along the bottom of the pipe before returning to the reservoir. The aeroponics units (Figure 6B) used the same pipe–reservoir architecture, but the pipes were mounted vertically to create an open root chamber. The nutrient solution was delivered from the top of the vertical pipe, allowing the solution to trickle downward towards the roots. Excess solution was collected at the bottom of the pipe and returned to the reservoir. In both systems, each pipe included seven planting holes (55 mm diameter) spaced at 200 mm intervals.

In each system, three of the four subsystems were equipped with 350 W heating elements installed in the reservoirs, allowing for temperature increase of the nutrient solution. Temperature regulation relied on DS18B20 sensors (Analog Devices Inc./Maxim Integrated, Wilmington, MA, USA) connected to an Arduino Uno microcontroller, which activated heating elements via relays to maintain temperature above the target values. A separate data-logging system recorded the nutrient solution temperatures inside each hydroponic pipe. Ambient temperature was also monitored (MX1104 HOBO Data Logger, Cape Cod, MA, USA) and is presented in Table 1. Air relative humidity was 55 ± 10%.

Root-zone temperature was controlled indirectly through precise regulation of the minimum nutrient solution temperature in the reservoirs supplying each sub-system. The reported temperatures refer to the nutrient solution at the point of delivery to the growing units [15,26]. In both soilless systems, we tested the control treatment with no temperature adjustments, as well as minimum root-zone temperatures of 14, 18, and 22 °C. According to the above, the NFT treatments are hereby labeled as NFT-C, NFT-14, NFT-18, and NFT-22, while the aeroponics treatments are hereby labeled as Aero-C, Aero-14, Aero-18, and Aero-22. Due to natural diurnal fluctuations, the actual root-zone temperatures were higher and are shown in Table 1. Within a soilless system, each root-zone temperature treatment included one subsystem containing seven plants in the NFT or eight plants in aeroponics. In addition, each temperature treatment was applied to a single independent cultivation unit, where individual plants were considered replicates since all plants were exposed to identical conditions. Statistical analyses were conducted within each soilless system, while avoiding direct comparisons between the soilless systems.

The two soilless systems were selected because of the differences they provide in the root environment related to the temperature control. The NFT is nowadays the most employed hydroponic system throughout the world, while aeroponics is promoted as a novel approach, with increasing research interest due to the constant root exposure in the air. In addition, these temperature regimes (14, 18, and 22 °C) were selected to test a range of realistic winter conditions taking place in the Mediterranean region.

The nutrient solution composition was obtained by Nutrisense Decision Support System (https://nutrisense.online/; accessed on 5 December 2023), specialized software used to calculate nutrient concentrations in various crops [36]. Briefly, electrical conductivity (EC) was 2.0 dS/m, pH was 5.6 (adjusted with nitric acid 68%), NO_3_^−^ was 11.6 mM, NH_4_^+^ was 1.6 mM, K^+^ was 6.4 mM, Ca^2+^ was 4.7 mM, Mg^2+^ was 1.7 mM, H_2_PO_4_^−^ was 1.1 mM, and Fe was 16.0 μM. The solution also contained trace elements (i.e., Mn^2+^, Zn^2+^, Cu^2+^, B, and Mo). The nutrient solution was prepared using fertigation-grade fertilizers (water-soluble salts suitable for hydroponic application), dissolved directly into tap water. The water used for solution preparation had an electrical conductivity of 0.33 dS/m and a pH of 7.8. The nutrient solution was replenished weekly due to plant consumption, as well as to keep EC and pH at the desired levels. Mixing of the nutrient solution was ensured through continuous pumping and recirculation in the reservoirs. All nozzles were inspected regularly to ensure that no clogging occurred.

### 4.2. Measurements and Analyses

Plants were harvested after 29 days of cultivation. Measurements included a range of biomass-related, morphological, physiological, nutritional, and safety-related characteristics to properly describe the overall plant development under the effect of the different treatments. Specifically, leaf fresh weight was measured to calculate the LMSA depending on the planting density of each soilless system (i.e., 15.2 plants/m^2^ in the NFT and 22.2 plants/m^2^ in aeroponics). Root dry weight (after 3 days in a drying oven at 72 °C) and maximum leaf length were also measured. Relative anthocyanin content was recorded with an ACM-200+ (Opti-Sciences, Hudson, NH, USA), which provides an anthocyanin index based on leaf optical properties. Chlorophyll fluorescence parameters (PI_abs_ and PI_tot_) were recorded with Pocket PEA (Hansatech Instruments, Amesbury, MA, USA).

Later, leaves were homogenized and placed in a freezer (−20 °C) for analysis of the nutritional content (3 replications per treatment). Total soluble solids (Brix°) were recorded with a PAL-α refractometer (Atago, Tokyo, Japan). Total phenolic content was quantified with the Folin–Ciocalteu assay [37] upon extraction with 80% methanol. Absorbance of the colored reagent was measured with a spectrophotometer at 760 nm. Antioxidant activity was quantified with the ferric-reducing antioxidant power (FRAP) assay [38] upon extraction with 80% methanol. Absorbance of the colored reagent (sample with CH_3_COONa, TPTZ, and FeCl_3_) was measured with a spectrophotometer at 593 nm. Photosynthetic pigments (chlorophylls and carotenoids) were quantified upon extraction with 80% aqueous acetone [39]. Absorbance of the colored acetone was measured with a spectrophotometer at 470, 647, and 663 nm. Finally, nitrate content was quantified after extraction in 25 mL water [40]. Absorbance of the colored reagent (sample, H_2_SO_4_, and NaOH) was measured with a spectrophotometer at 410 nm.

### 4.3. Statistical Analysis

Analysis of variance (ANOVA) was conducted with SPSS 25.0 (IBM Corp., Armonk, NY, USA) software. Post-hoc comparisons between temperatures and within soilless systems was performed with Tukey’s HSD method at significance level α = 0.05.

## 5. Conclusions

Our findings highlight that controlling root-zone temperature is a valuable tool for the optimization of the growth and development of mini Romaine lettuce, especially in NFT systems. A minimum root-zone temperature of 22 °C led to increased yield and a greater amount of total soluble solids, but a lower pigment content and photosynthetic capacity (though plants were healthy) compared to the lowest temperature regimes. This can be attributed to the lowest ΔΤ recorded among all treatments. In our aeroponics system, produced yield was positively affected by increased temperature regimes, but differences were less profound regarding the physiological and nutritional profile. Romaine lettuce exhibited limited capacity to sustain root growth at suboptimal root-zone temperatures, despite preserved photosynthetic activity, showing that growth limitation is primarily due to sink-related characteristics rather than carbon assimilation. Compared with the optimal root-zone temperature, lower temperatures resulted in reduced growth efficiency, highlighting the narrow thermal tolerance window of romaine lettuce during soilless cultivation. Overall, the production efficiency of soilless systems can be enhanced by heating the nutrient solution, especially in low- and mid-tech greenhouses during cool months with a view to reduced ΔT. The utilization of such knowledge can contribute significantly to the sustainable production of fresh leafy vegetables in such systems and climates, and even in future climate scenarios where extreme temperature fluctuations are predicted. The variable responses manifested by the two soilless systems emphasize the necessity for further research to determine the optimum temperature regimes for each crop species and even genotype.

## Figures and Tables

**Table 1:** Daily average, maximum, and minimum temperatures monitored inside the nutrient solution reservoir of each treatment, as well as in the surrounding environment (air). NFT: nutrient film technique. Aero: aeroponics. C: control.

Treatment	Average (°C)	Mean Maximum (°C)	Mean Minimum (°C)
NFT-C	16.7	22.2	12.2
NFT-14	17.3	22.1	14.4
NFT-18	20.7	23.2	18.6
NFT-22	23.8	25.4	22.2
Aero-C	16.1	22.6	10.9
Aero-14	17.8	22.8	14.0
Aero-18	21.2	24.8	18.2
Aero-22	24.9	26.5	22.5
Air	15.8	26.2	8.4

## Data Availability

The original contributions presented in this study are included in the article. Further inquiries can be directed to the corresponding author.
